# Acaricidal Activity of Bufadienolides Isolated from *Drimia pancration* against *Tetranychus urticae,* and Structural Elucidation of Arenobufagin-3-*O-α*-L-rhamnopyranoside

**DOI:** 10.3390/plants11131629

**Published:** 2022-06-21

**Authors:** Natale Badalamenti, Maurizio Bruno, Roman Pavela, Filippo Maggi, Oliviero Marinelli, Laura Zeppa, Giovanni Benelli, Angelo Canale

**Affiliations:** 1 Dipartimento di Scienze e Tecnologie Biologiche, Chimiche e Farmaceutiche (STEBICEF), Università degli Studi di Palermo, Viale delle Scienze, 90128 Palermo, Italy; natale.badalamenti@unipa.it (N.B.); maurizio.bruno@unipa.it (M.B.); 2 Centro Interdipartimentale di Ricerca, Riutilizzo Bio-Based Degli Scarti da Matrici Agroalimentari (RIVIVE), Università di Palermo, Viale delle Scienze, 90128 Palermo, Italy; 3 Crop Research Institute, Drnovska 507, 161 06 Prague, Czech Republic; pavela@vurv.cz; 4 Department of Plant Protection, Czech University of Life Sciences Prague, Kamycka 129, 165 00 Prague, Czech Republic; 5 Chemistry Interdisciplinary Project (ChIP), School of Pharmacy, University of Camerino, Via Madonna delle Carceri 9/B, 62032 Camerino, Italy; 6 School of Pharmacy, University of Camerino, Via Madonna delle Carceri 9, 62032 Camerino, Italy; oliviero.marinelli@unicam.it (O.M.); laura.zeppa@unicam.it (L.Z.); 7 Department of Agriculture, Food and Environment, University of Pisa, Via del Borghetto 80, 56124 Pisa, Italy; giovanni.benelli@unipi.it (G.B.); angelo.canale@unipi.it (A.C.)

**Keywords:** botanical acaricide, crop pest, Integrated Pest Management, steroidal saponins, NMR

## Abstract

Chemical characterization of the bulbs of *Drimia pancration* was conducted to isolate four steroidal saponins (**1**–**4**). Earlier, we focused on the structural elucidation of compounds **1**–**3**. Herein, by means of ^1^H-NMR, ^13^C-NMR, Nuclear Overhauser Effects (NOE), and 2D-NMR spectra, the full stereochemical structure of **4** is reported, and all the ^1^H and ^13^C signals are assigned. Compounds **1**–**4** were tested for their acaricidal properties against the two-spotted spider mite *Tetranychus urticae*. Our results showed excellent activity of compound **1,** with an LD_50_ (µg/cm^2^) of 0.29 and a LD_90_ (µg/cm^2^) of 0.96, whereas compounds **2**, **3**, and **4** showed moderate activity. Furthermore, the acaricidal and cytotoxic properties of the crude extract were also investigated. Of note, after 96 h of exposure, the acaricidal activity of compound 1 was higher than that of the positive control, hexythiazox. Indeed, for compound **1**, LD_50_ and LD_90_ were 0.29 and 0.96 µg/cm^2^, respectively, while hexythiazox LD_50(90)_ was 18.7 (132.5) µg/cm^2^. Additionally, *D. pancration* extract, after 72 h, induced a high cytotoxic effect in HaCaT and THP-1 cell lines, with an IC_50_ of 7.37 ± 0.5 µg/mL and 3.50 ± 0.15 µg/mL, respectively. Overall, *D. pancration* can be considered as a green source of novel acaricides effective against mites of agricultural importance, such as *T. urticae*, pending proper field validation and the assessment of non-target effects on other invertebrate species.

## 1. Introduction

Managing insect and mite pests of agricultural importance is a major challenge nowadays, considering the quick and widespread development of pesticide resistance in overexposed pest populations [1], as well as the major impact of pesticide use on human health and the environment [2,3,4]. Among mites, the two-spotted spider mite, *Tetranychus urticae* Koch (Arachnida: Acari: Tetranychidae) is an excellent example of a species with huge economic importance coupled with the ability to quickly develop resistance to several classes of chemical acaricides [5,6], which is boosted by high fecundity, inbreeding, arrhenotokous reproduction, and short life cycle [7,8]. Of note, *T. urticae* can attack more than 1100 host plants, including both greenhouse and open-field crops [9,10].

In this scenario, plants represent a promising reservoir of secondary metabolites with insecticidal [11] and acaricidal activity [12,13,14,15], characterized by a multiple mode of action that reduces the likelihood of resistance development [16,17].

*Drimia pancration* (Steinh.) J. C. Manning & Goldblatt (syn. *Scilla pancration* (Steinh.) Nyman, *Urginea maritima* subsp. *pancration* (Steinh.) K. Richt., *Squilla pancration* Steinh., *Charybdis pancration* (Steinh.) Speta), belonging to the family Asparagaceae, is a bulb distributed across Africa (Morocco, Libya, Algeria), Italy (including Sicily, Malta, Baleares), and Corsica [18].

It is very similar to *Drimia maritima* (L.) Stearn, commonly referred to as sea squill or sea onion, a species absent from Sicily, that has been largely investigated due to its important biological properties, such as cardiotonic and diuretic properties; for heart disease and oedema [19], much like rodenticide [20], and for insecticidal activity against *Drosophila melanogaster* Meigen [21]. The large number of studies published on the phytochemistry of *D. maritima* showed the occurrence of cardiac glycosides [22,23], anthocyanins [24], lignans [25], flavonoids, fatty acids, and polysaccharides [24], which have been reviewed by some authors [26].

On the other hand, few studies have been conducted on *D. pancration*. Two papers reported the identification of different bufadienolides obtained from the roots of two different populations collected from Southern Italy. [27,28]. Notably, in a recent investigation on a Sicilian accession [29], the authors isolated (2*R*,3*R*)-dihydrokaempferol 3-*O*-*β*-D-glucoside, and three steroidal saponins: (5*α*)-4,5-dihydro-16β-hydroxyscillirosidin-3-*O*-*α*-L-thevetopyranoside (**1**), scilliglaucoside (**2**), and (5*α*)-4,5-dihydrosicillirosidin-3-*O*-(*β*-D-glucopyranosyl-(1→4)-*α*-L-thevetopyranoside) (**3**). Furthermore, in the same paper, the insecticidal properties of the methanol and butanol extracts of *D. pancration* were proven effective against adult *Stegobium paniceum* beetles [29].

Consequently, in the frame of our ongoing research on Sicilian plants [30,31,32,33], and possible biocidal applications [29,34,35,36], inspired by the insecticidal activity shown by both the extracts of *D. pancration* and by the cardiac glycosides [29,37], we decided to re-investigate the presence of bufadienolides in a *D. pancration* population collected near Palermo, Sicily, and to test the acaricidal properties of the pure isolated compounds on the two-spotted spider mite, *T. urticae*. 

## 2. Results and Discussion

### 2.1. Chemical Compounds

**Figure 1:**
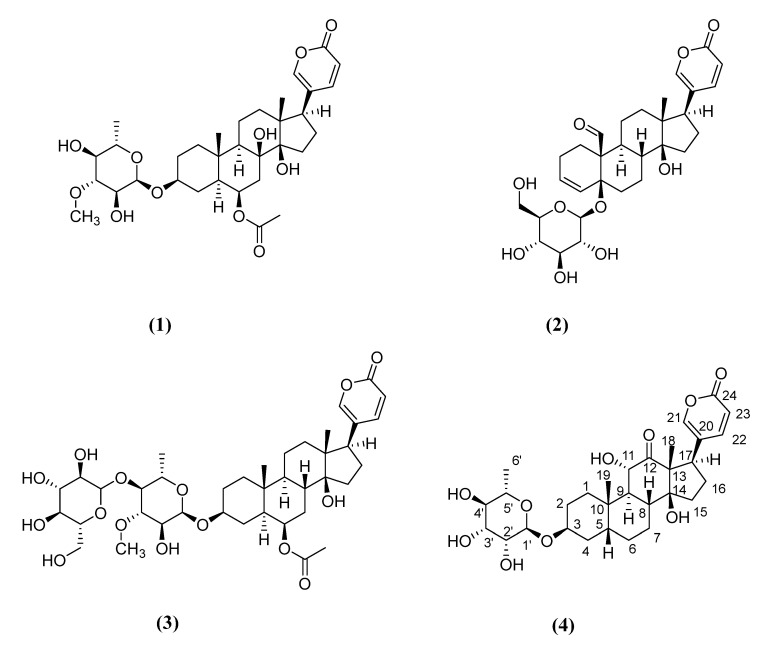
Chemical structures (**1**–**4**) of bufadienolides isolated from *D. pancration*.

The four steroidal saponins (**1**–**4**) (Figure 1) were isolated, by different chromatographic separations, from the bulbs of *D. pancration*, extracted in *n-*butanol.

In a previous paper, we reported the structures of compounds **1**–**3** [29]; consequently, in the present communication, only the structural elucidation of compound **4**, determined by 1D- and 2D-NMR, and HPLC–MS spectra, is highlighted. 

Compound **4** was separated into white plates. HPLC–MS (Figure S1) showed a molecular ion at *m/z* 585.2513 [M+Na]^+^ (calcd. for 585.2675), in agreement with the molecular formula of C_30_H_42_O_10_. The ^1^H-NMR and ^13^C-NMR (Table S1) spectra (Figures S2 and S3) showed signals for an unsaturated *α,β,γ,δ*-lactone at *δ*H = 7.55 (H-21), 7.79 (H-22), 6.30 (H-23) ppm, and at *δ*C = 121.29 (C-20), 150.47 (C-21), 147.53 (C-22), 114.89 (C-23), and 161.71 (C-24) ppm, two angular methyl groups (*δ*H = 0.77 ppm, 3H, s; *δ*C = 17.60 ppm, Me-18), and (*δ*H = 1.04 ppm, 3H, s; *δ*C = 23.79 ppm, Me-19), two oxygenated methines at C-11 (*δ*H = 4.24 ppm, d; *δ*C = 73.61 ppm) and C-3 (*δ*H = 3.43 ppm, m; *δ*C = 71.03 ppm), a keto group C-12 (*δ*C = 213.73 ppm), and a quaternary oxygenated carbon (*δ*C = 84.51 ppm, C-14). Furthermore, signals of an *α-*L-rhamnopyranoside sugar moiety were observed. 

Using DEPT, ^1^H-^1^H COSY, HSQC, and HMBC spectra, the complete plane structure of compound **4** was identified. Indeed, the HMBC spectrum correlation between the anomeric proton H_1′_ (*δ*H = 4.59 ppm, d, *J* = 1.5 Hz) and the aglycone C-3 (*δ*C = 71.03 ppm) clearly indicated that the sugar moiety was linked at C-3. 

**Figure 2:**
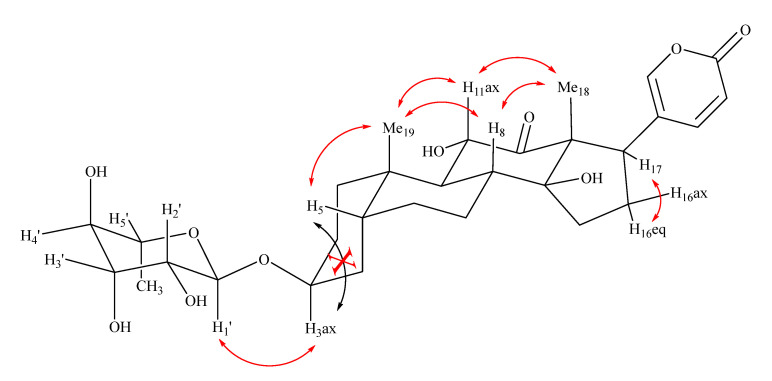
NOESY correlations in compound **4**.

Finally, the NOESY correlation (Figure 2) of Me-19/H-5*β*, Me-19/H-11_ax_, H-11_ax_/Me-18, H-17_ax_/H-16_eq_, and the absence of correlation H-3/H-5 not only confirmed the *β* orientation of the *O*-C-3 glycosyl group, but also the correct junction between the aglycon rings: *cis* between the A/B and C/D rings, and *trans* between the B/C rings. Consequently, the structure of **4** was established as arenobufagin-3-*O-α*-L-rhamnopyranoside, previously isolated in *D. altissima* [38], but not in *D. pancration*.

### 2.2. Acaricidal Effect

All compounds tested here, as well as the extract, showed promising acaricidal activity (Table 1). However, significant differences in acaricidal efficacy between the individual substances were found. The most effective substance was compound **1**, which, at a dose of 100 µg/cm^2^, was the only one to cause 100% mortality within 24 h of application. Other substances tested by us, including the crude *D. pancration* extract, showed significant mortality up to 96 h after application, with mortality rates exerted by compound **1** comparable to those triggered by the positive control, hexythiazox. 

Lethal doses were estimated for all substances except **3**, which showed low acaricidal efficacy. Significantly, the lowest LD_50_ and LD_90_ values were estimated for **1** (0.29 and 0.96 µg/cm^2^, respectively). This compound showed significantly higher efficacy than the tested commercial acaricide based on hexythiazox, for which the LD_50(90)_ was estimated to be 18.7 (132.5) µg/cm^2^.

Given that a number of resistant populations of *T. urticae* has already emerged [6,39], there is an urgent need to look for new active substances with acaricidal activity. Plant extracts are among the most promising sources of compounds with a novel mechanism of action [40]. In particular, the compound **1** isolated by the present authors seems to be a highly promising candidate for the synthesis of new acaricide substances due to its high acaricidal activity. However, further testing will be needed to reveal the mechanism of action and the effect of this substance on non-target organisms.

### 2.3. Cytotoxicity Assay

**Figure 3:**
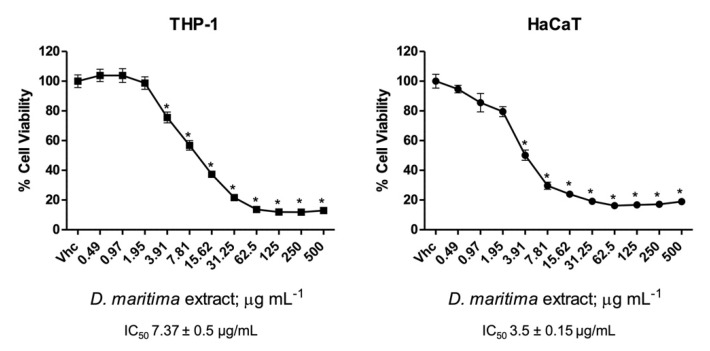
*Drimia pancration* extract cytotoxic effect: cell viability was determined in THP-1 and HaCaT cell lines by MTT assay. Cells were treated for 72 h with different extract concentrations. Data are expressed as mean ± SD of three separate experiments. * *p* < 0.05 vs. vehicle (Vhc).

Monocytic cell line THP-1 and immortalized human keratinocyte cell line (HaCaT) were treated with different dilutions (from 0.49 up to 500 µg/mL) of *D. pancration* extract for 72 h, and cell viability was assessed by cytotoxic assay. As reported in Figure 3, the total extract induced a cytotoxic effect in both cell lines, with an IC_50_ of 7.37 ± 0.5 µg/mL and 3.50 ± 0.15 µg/mL, for THP-1 and HaCaT cells, respectively. According to the International Organization for Standardization guidelines, the total extract showed a high cytotoxicity, since it caused > 70% of cell mortality when it was used at 100 μg/mL [41].

## 3. Materials and Methods

### 3.1. Plant Material

Prof. Vincenzo Ilardi, a botanist of the University of Palermo, collected samples in Cinisi, Palermo, Italy, and identified, in February 2020, the bulbs of *D. pancration*. The voucher deposited at the STEBICEF Department, University of Palermo, Italy, is identified by the code PAL266/2020.

### 3.2. Extraction, Isolation, and General Experimental Procedures

The extraction procedure, the isolation of the single metabolites, and all materials used (chemical reagents and laboratory tools), are the same as described in Badalamenti et al. [29]. Compound **4** was isolated from the butanol portion of the methanol extract of *D. pancration* roots. The fraction A13 (200 mg) was obtained by column chromatography with CHCl_3_-MeOH (7:3) to produce compound **4** (22 mg). Compounds **1**–**3** were previously described in [29].

#### Arenobufagin-3-*O*-*α*-L-rhamnopyranoside (**4**)

White plates; for proton and carbon chemical shifts. See Table S1; ESIMS *m*/*z* 585.2513 [M+Na]^+^ (calcd. for 585.2675).

### 3.3. Mites

*T. urticae* adults were obtained from the mass-rearing site established at the Crop Research Institute (Czech Republic). The mites were reared on bean plants in a growth chamber (22–25 °C; 16:8 h (L:D) photoperiod).

### 3.4. Acaricidal Activity

The acaricidal efficacy of *D. pancration* methanolic extract and isolated bufadienolides was measured as *T. urticae* adult mortality after 24 and 96 h of exposure [42]. Experiments were performed using bean leaf discs (*Phaseolus vulgaris* L.) sized 1 cm^2^. The extract and compounds were dissolved in methanol (p.a. 99%, Sigma-Aldrich, Czech Republic) at a dose of 1 mg in 100 µL of MeOH using an automatic pipette; aliquots (10 µL) of the methanolic solutions, containing a defined amount of *D. pancration* extract or compounds, were applied onto the leaf discs. This provided a dose of 100 µg/cm^2^. For the estimation of lethal doses alone, tests were performed with the following doses: compound **1**—0.1, 0.3, 0.5, 0.8, and 1.0 µg/cm^2^; for substance **2**—0.5, 1.0, 2.5, 5.0, 10.0, 20.0, 50.0, 80.0, 100.0 and 120.0 µg/cm^2^ (5 doses were selected for calculation); for substance **4**—12.5, 25.0, 50.0, 100.0, 200.0 and 400.0 mg/cm^2^; for extract—6.2, 12.5, 25.0, 50.0 and 100.0 µg/cm^2^. After that, the discs were placed in Petri dishes (5 cm) with an agar layer of 0.3 cm thick on the bottom. Only methanol was applied to the negative control discs. As a positive control, a commercial acaricide based on the active substance hexythiazox (Nissorun 25 SC, a.i. 250 g/L, registrant Nisso Chemical Europe GmbH) was used. Of note, this acaricide is classically used to target eggs and nymphs of *T. urticae*, and that a significantly higher dose is needed on adults. However, it has been reported to show some efficacy on adults. For example, Marris [43] (1988) showed that toxicity of hexythiazox on *T. urticae* females was 1.5 a.i. g/L, and Havasi et al. [44] (2021) reported a LC_50_ of 2.35 g/L on *T. urticae* adults.

After solvent evaporation, 10 *T. urticae* females were moved on the leaf disc sides treated with the compounds using a fine brush. Petri discs were inserted into a growth chamber (16:8 (L:D), 25 °C). Then, leaf discs were checked for the number of dead mites 24 and 96 h post-application. Mite mortality was recorded when the females did not react to forceps stimuli. Each experiment was repeated 5 times. For substances that showed a mortality of more than 50% at 96 h after application, a concentration series of 5 dilutions was subsequently created, which showed a mortality in the range of 10 to 90%. The range of concentrations was chosen for each substance based on preliminary experiments.

### 3.5. Cytotoxicity on Vertebrate Cells

Monocytic cell line THP-1 and immortalized human keratinocytes cell line (HaCaT) were provided by IFOM (Institute of Molecular Oncology, Rome, Italy). HaCaT cells were cultured in Dulbecco’s Modified Eagle Medium (DMEM) enriched with 10% fetal bovine serum (FBS), 100 IU/mL penicillin/streptomycin, and 2 mM L-glutamine, and kept at 37 °C with 5% CO_2_ and 95% humidity. 

Cytotoxicity was evaluated by adding 3-(4,5-dimethylthiazol-2-yl)-2,5 diphenyl tetrazolium bromide (MTT). Briefly, as previously described [45], 3 × 10^3^ cells per well were seeded in a 96-well plate at a final volume of 100 μL/well and, after 24 h of incubation, different dilutions of *D. pancration* extract were added and 6 replicates were used for each treatment. The effect was compared with dimethyl sulfoxide (DMSO) used to solubilize the extract. After 72 h, the cell viability was investigated by adding 0.8 mg/mL of MTT salt (Sigma Aldrich, Milan, Italy) to the media. After 3 h, the salt crystals were dissolved in 100 μL/well of DMSO. An ELISA reader microliter plate (BioTek Instruments, Winooski, VT, USA) was used to measure the absorbance of samples at 570 nm against a control.

### 3.6. Statistical Analysis

Experimental mite mortality rates lower than 20% compared to control were corrected via Abbott’s formula [46], and then Probit analysis was carried out [47]. Mite mortalities at 24 and 96 h were arcsine square root transformed and analyzed using ANOVA within a randomized complete block design, followed by Tukey’s HSD test (*p* < 0.05). The obtained data were analyzed using the software BioStat v5.0. 

The data for cell cytotoxicity represent the mean and standard deviation (SD) of at least 3 independent experiments. The statistical significance was determined by one-way ANOVA with Bonferroni’s post hoc test; *α* was set at 0.05. IC_50_ was calculated using GraphPad Prism software [48].

## 4. Conclusions

In this chemical study on the bulbs of *D. pancration*, by mean of 1D- and 2D-NMR, NOESY, and HPLC–MS spectra, the full stereochemical structure of arenobufagin-3-*O-α*-L-rhamnopyranoside (**4**) was revealed. This bufadienolide, together with the other three (**1**–**3**) previously isolated compounds, was evaluated for its potential activity as an acaricide. From a green pesticide point of view, *D. pancration,* despite the fact that the total extract shows a high cytotoxicity, can be used in formulation, to mitigate its cytotoxicity, or considered as a green source of novel acaricides—with special reference to compound **1**—effective against mites of agricultural importance, such as *T. urticae*, pending proper field validation and the assessment of non-target effects on other invertebrate species.

## Figures and Tables

**Table 1:** Acaricidal effect of the isolated compounds and *Drimia pancration* extract against *Tetranychus urticae* females 96 h after application.

Tested Acaricide	Mortality **		Lethal Dose (for 96 h)			
	At 24 h (Dose 100 µg/cm^2^)	At 96 h (Dose 100 µg/cm^2^)	LD_50_ (µg/cm^2^)	LD_90_ (µg/cm^2^)	χ^2^	*p*-Value
1	100.0 ± 0.0e	100.0 ± 0.0d	0.28 (0.22–0.34)	1.08 (0.84–1.60)	1.485	0.685
2	47.4 ± 11.7c	83.3 ± 4.7c	1.41 (0.62–2.48)	198.58 (86.01–786.52)	1.836	0.968
3	16.4 ± 5.6b	46.7 ± 12.5b	˃100			
4	10.6 ± 5.4ab	70.0 ± 8.2c	29.61 (13.20–48.35)	1862.15 (648.5–2651.24)	0.407	0.981
*D. pancration* extract	5.5 ± 2.5a	78.9 ± 6.3c	8.5 (5.9–10.6)	118.8 (103.3–129.5)	0.852	0.384
Positive control Hexythiazox	82.5 ± 12.5d	100.0 ± 0.0d	18.7 (12.7–21.5)	132.5 (111.7–142.8)	1.234	0.251
Negative control	0.0 ± 0.0a	6.7 ± 4.7a				
ANOVA F_6,28_, *p*-value *	381.72; 0.000	273.57; 0.000				

* ANOVA parameters; ** means followed in the same column by the same letter are not significantly different (ANOVA, Tukey’s HSD test, *p* < 0.05).

## Data Availability

Not applicable.

## Supplementary Materials

The following supporting information can be downloaded at: https://www.mdpi.com/article/10.3390/plants11131629/s1, Table S1: ^1^H-NMR and ^13^C-NMR of compound **4** (*δ* in ppm); Figure S1. Mass spectrum of compound **4**; Figure S2. ^1^H-NMR spectrum of compound **4** (δ in ppm); Figure S3. ^13^C-NMR spectrum of compound **4** (δ in ppm).
