# Supplementary material for: Acaricidal Activity of Bufadienolides Isolated from Drimia pancration against Tetranychus urticae, and Structural Elucidation of Arenobufagin-3-O-α-L-rhamnopyranoside
Source: Plants (Basel). 2022 Jun 21;11(13):1629. doi: 10.3390/plants11131629 (PMC9268777; doi:10.3390/plants11131629)
Supplement: Supplementary file 1 [file plants-11-01629-s001.zip › plants-1733089-supplementary.pdf]

plants

Supplementary Materials

Table S1. Proton and carbon chemical shifts of compound **4** (*δ* in ppm).

Position	δ_C[a]	δ_H[a] (*J* in Hz)
1*β*	32.17	1.23 (m)
1*α*		1.62 (m)
2*α*	26.70	1.18 (m)
2*β*		1.79 (m)
3*α*	71.03	3.43 (m)
4*β*	30.22	1.30 (m)
4*α*		1.62 (m)
5*β*	40.05	2.48 (dddd, 1.8, 1.8, 1.8, 1.8)
6*α*	27.92	1.37 (m)
6*β*		1.79 (m)
7*α*	21.61	1.18 (m)
7*β*		1.82 (m)
8	39.92	1.68 (m)
9*α*	39.56	1.71 (m)
10	36.80	-
11*α*	73.61	4.24 (d, 11.2)
12	213.73	-
13	62.48	-
14	84.51	-
15*β*	32.74	2.06 (m)
15*α*		2.36 (m)
16*β*	27.93	1.60 (m)
16*α*		1.83 (m)
17*α*	40.40	3.94 (dd, 9.4, 7.0)
18	17.60	0.77 (s)
19	23.79	1.04 (s)
20	121.29	-
21	150.47	7.55 (dd, 2.5, 1.0)
22	147.53	7.79 (dd, 9.8, 2.5)
23	114.89	6.30 (dd, 9.8, 1.0)
24	161.71	-
1′	98.44	4.59 (d, 1.5)
2′	71.48	3.54 (m)
3′	71.83	3.76 (m)
4′	72.35	3.18 (dd, 9.0, 9.0)
5′	68.89	3.41 (m)
6′	18.13	1.07 (d, 6.3)

[a] $(CD_3)_2SO$ (^1H: 400 MHz; ^{13}C: 100 MHz).

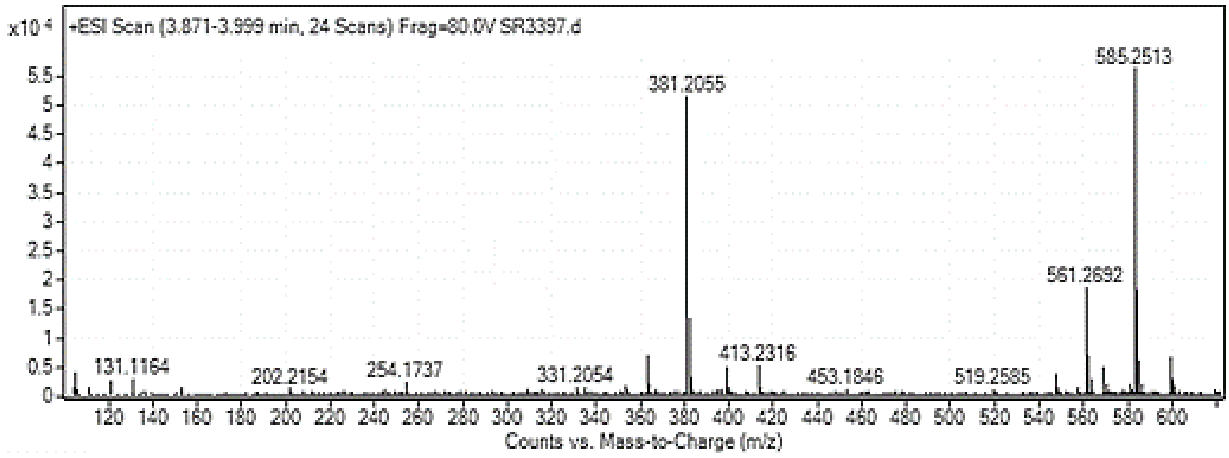

Figure S1. Mass spectrum of compound **4**.

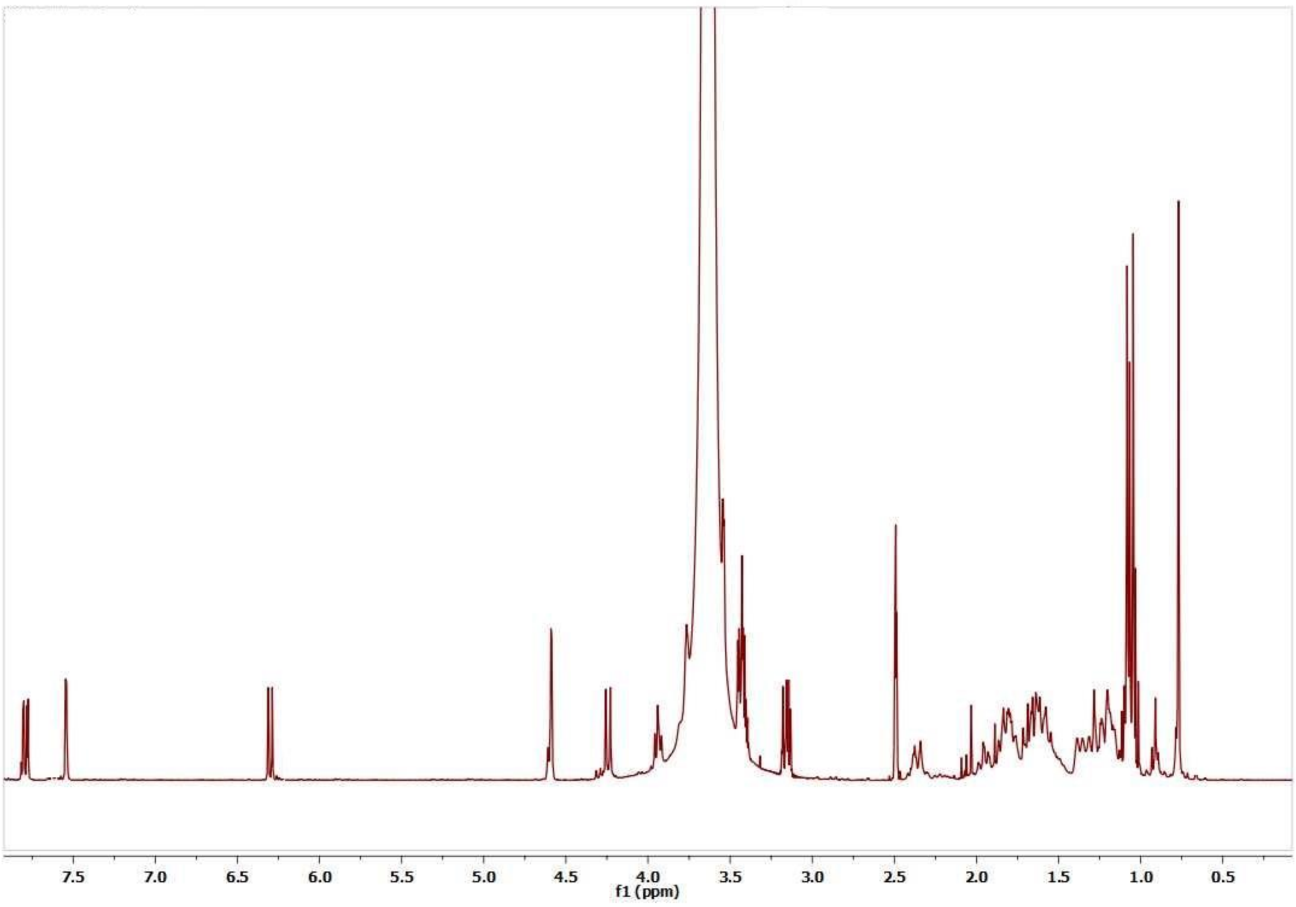

Figure S2. ^{1}H-NMR spectrum of compound **4** (δ in ppm).

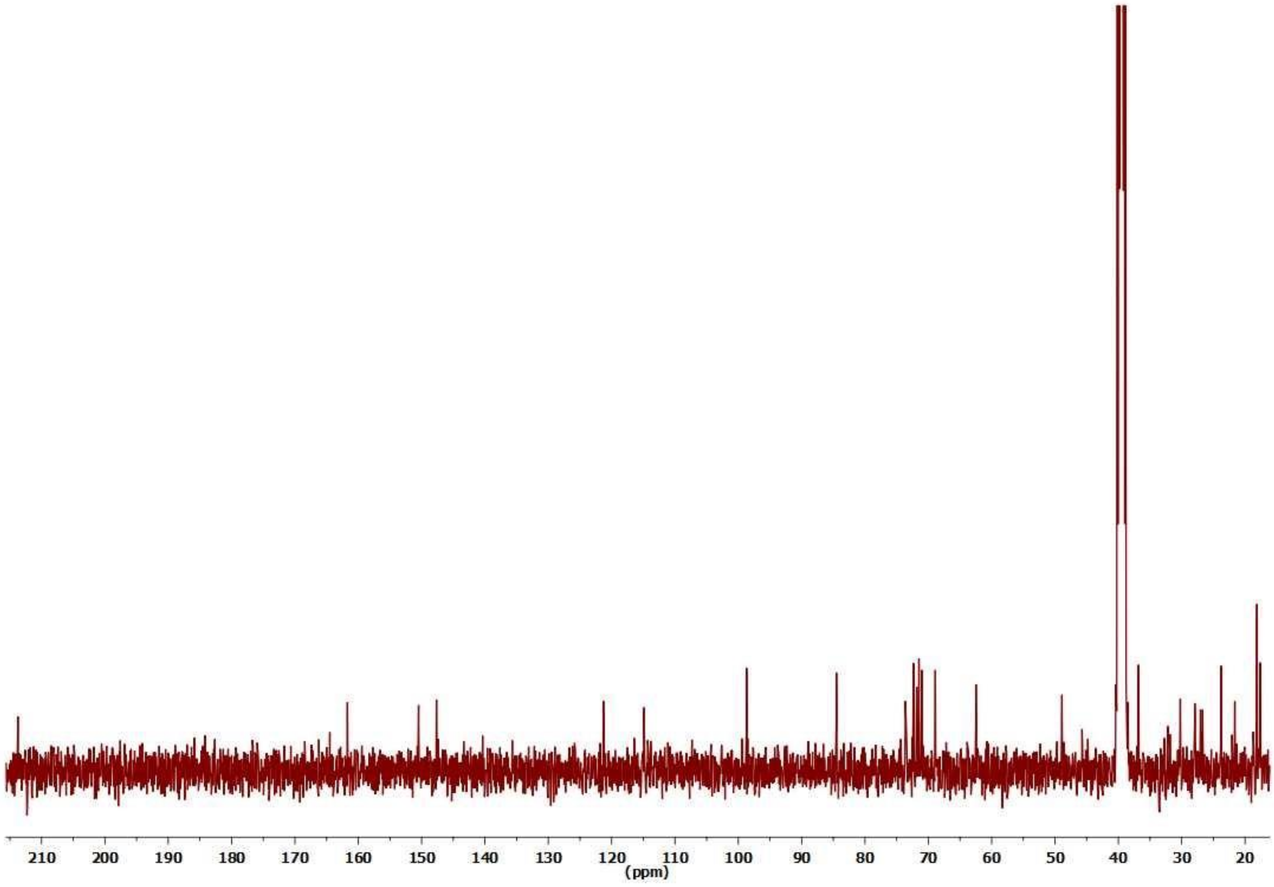

Figure S3. ^{13}C-NMR spectrum of compound **4** (δ in ppm).
